# Molecular Pattern and Density of Axons in the Long Head of the Biceps Tendon and the Superior Labrum

**DOI:** 10.3390/jcm8122129

**Published:** 2019-12-03

**Authors:** Sandra Boesmueller, Roland Blumer, Bernhard Gesslbauer, Lena Hirtler, Christian Fialka, Rainer Mittermayr

**Affiliations:** 1 AUVA Trauma Center Vienna Meidling, Kundratstraße 37, 1120 Vienna, Austria; christian.fialka@auva.at (C.F.); rainer.mittermayr@auva.at (R.M.); 2 Center for Anatomy and Cell Biology, Medical University of Vienna, Währinger Straße 13, 1090 Vienna, Austria; roland.blumer@meduniwien.ac.at (R.B.); lena.hirtler@meduniwien.ac.at (L.H.); 3 Department of Surgery, Division of Plastic and Reconstructive Surgery, Medical University of Vienna, Währinger Gürtel 18-20, 1090 Vienna, Austria; bernhard.gesslbauer@meduniwien.ac.at; 4 Faculty of Medicine, Sigmund Freud University, Freudplatz 1, 1020 Vienna, Austria; 5 Ludwig Boltzmann Institute for Experimental and Clinical Traumatology, Donaueschingen Straße 13, 1200 Vienna, Austria

**Keywords:** axons, pain, long head of the biceps tendon, superior labrum, SLAP

## Abstract

The type II superior labrum anterior to posterior (SLAP) repair is a viable option in young and demanding patients, although a prolonged period of pain after surgery is described in the literature. The reason for this fact remains unknown. Thus, the purpose of this study was to investigate the molecular pattern of the biceps tendon anchor, where the sutures for repair are placed. The long head of the biceps tendon (LHBT), including the superior labrum, was dissected in the setting of reverse total shoulder arthroplasty. Immunohistochemical staining was performed using neurofilament (NF) and protein gene product (PGP) 9.5 as general markers for axons and calcitonin gene-related peptide (CGRP) and substance P for nociceptive transmission. A quantitative assessment was performed according to the two regions of interest (ROIs), i.e., the anterosuperior (ROI I) and the posterosuperior labrum (ROI II). Eleven LHBTs with a mean age of 73 years (range: 66–87 years) were harvested intraoperatively. Six LHBTs were gained in osteoarthrosis and five in fractures. We found an inhomogeneous distribution of axons in the anterosuperior and posterosuperior parts of the labrum in all the specimens irrespective of the age, gender, and baseline situation. There was a significantly higher number (*p* < 0.01) as well as density (*p* < 0.001) of NF-positive axons in ROI I compared to ROI II. Nociceptive fibers were always found along the NF-positive axons. Thus, our results indicate that the biceps tendon anchor itself is a highly innervated region comprising different nerve qualities. The anterosuperior labrum contains a higher absolute number and density of axons compared to the posterosuperior parts. Furthermore, we were able to prove the presence of nociceptive fibers in the superior labrum. The results obtained in this study could contribute to the variability of pain after SLAP repair.

## 1. Introduction

One of the recommended surgical options for the type II superior labrum anterior to posterior (SLAP) lesion is arthroscopic repair using knotless suture anchors [1,2,3]. Although SLAP lesions represent a rare entity in the spectrum of shoulder injuries in younger patients, the number of repairs has tremendously increased in the first decade of this century [4,5]. Later on, the treatment priority changed toward biceps tenodesis as clinical results and the interval to return to sports activities, especially in overhead athletes as well as in patients aged 40 years or older, were not that promising [6,7,8,9,10,11]. The latest results even suggest that surgical treatment has no superiority over sham surgery or non-operative treatment [12,13,14]. Interestingly, recent editorial comments in high impact journals have pointed out that there still do not exist any guidelines regarding history, examination, diagnosis, and imaging of the SLAP lesion [15,16]. In addition, it is criticized that there are no criteria for successful SLAP repair and no consensus is available for surgical indications, characteristics, or rehabilitation [17]. This deficiency might even cause the impression that time has brought us back to the start.

Research focuses on clinical outcomes with no direct indications for or against a surgical technique [5,8,9,10,11,18]. Detrimental facts like persistent pain after SLAP repair is commonly described, but a possible explanation for this is still under investigation [6,9,19,20,21,22]. An increase in knowledge could be achieved by a more fundamental approach investigating the anatomical and histological features of the area of interest. As pain derives from unmyelinated axons, a few authors have already performed histological studies using neurofilament visualization to verify neural structures in the long head of the biceps tendon (LHBT) [23,24]. Furthermore, neurons have also been traced by neurofilament staining in the superior labrum, where suture anchors for SLAP repair are usually placed with an inhomogeneous density and distribution [25]. An evidence for differentiation into different nerve qualities has not yet been investigated in this area so far.

Thus, this study aims to prove that axons are present in the biceps tendon anchor comprising different nerve qualities, especially fibers responsible for the transmission of pain, by using different antibody staining. Since there is evidence that following SLAP repair, pain is more prominent when anchors are placed anterior and posterior versus posterior [26], we hypothesize that the neuronal structures display an unequal distribution with varying density in the anterosuperior as well as posterosuperior labrum.

## 2. Materials and Methods

**Figure 1:**
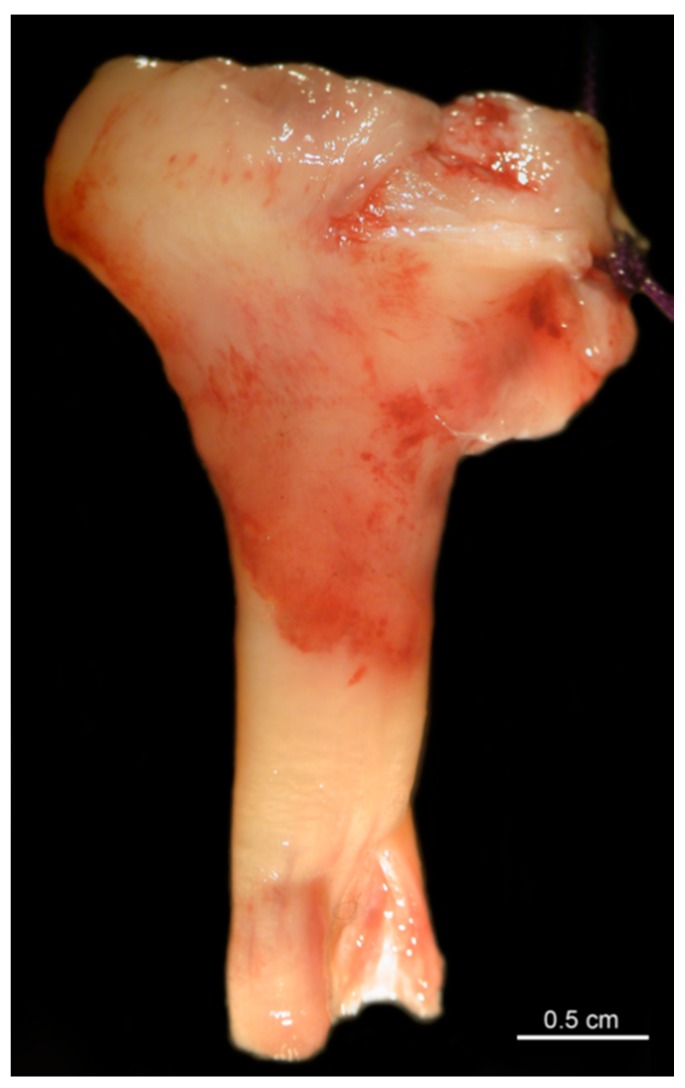
Macroscopic view of a dissected long head of the biceps tendon (LHBT), including the superior labrum.

The long head of the biceps tendon (LHBT), including the anterior and posterior parts of the superior labrum, was dissected intraoperatively in the setting of reverse total shoulder arthroplasty (RTSA) implanted either in osteoarthrosis (OA) or non-reconstructable Neer grade III or IV fractures (Figure 1). The anterosuperior labrum was marked with a suture to assure the correct orientation in the histological investigations. The inclusion criterion was a macroscopically intact long biceps tendon and its anchor in the superior labral region. Exclusion criteria were patients with a LHBT rupture, patients who had undergone previous biceps tenotomy, and patients with an apparent damage such as longitudinal ruptures or tendinosis. All tendons were assessed by the first (S.B.) and senior (R.M.) authors prior to histological investigations.

### 2.1. Immunohistochemistry

All specimens were fixed in 4.5% formaldehyde immediately after harvest, and immersion fixation was done for 24 h. Afterwards, they were kept in PBS buffer (pH 7.4, 0.1 M) for another 24 h. The specimens were cryoprotected in 40% sucrose, frozen, and kept at −80 °C until further processing. Transversal sections of 10 μm thickness were cut.

Immunofluorescence staining was performed on the frozen sections using several antibodies for different nerve qualities. As general markers for axons, we used chicken anti-neurofilament (Merck, Billerica, MA, USA; Cat# AB5539, RRID: AB_177520; 1:2000) and rabbit anti-protein gene product (PGP) 9.5 (Merck; Cat# NE1013, RRID: AB_2210632; 1:300). For nociceptive axon visualization, rabbit anti-CGRP (Merck; Cat# AB15360, RRID: AB_672958; 1:1000) and mouse anti-substance P (Abcam; Cat# AB14184, RRID: AB_300971; 1:500) were used. Four combinations of double-labeling were performed as follows: incubation with (i) antibodies against neurofilament and protein gene product (PGP) 9.5, (ii) antibodies against neurofilament and calcitonin gene-related peptide (CGRP), (iii) antibodies against neurofilament and substance P, and (iv) antibodies against CGRP and substance P [27]. Positive controls were performed in the human spinal cord obtained from an organ donor to test the validity of the antibodies used in the present study [28].

### 2.2. Detection and Quantification of Neurofilament (NF)-Positive Cells

**Figure 2:**
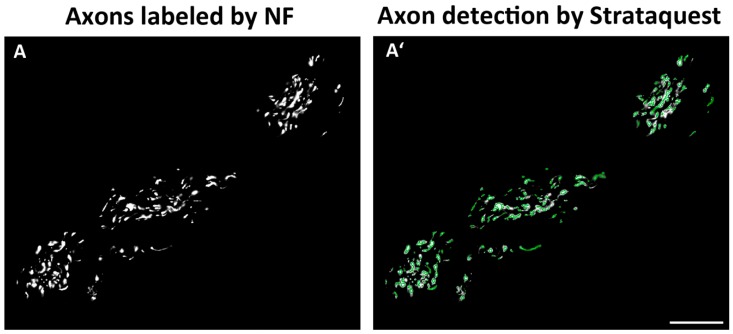
(**A**) shows axons immunolabeled with anti-neurofilament (NF) and (**A′**) the same axons following automatic detection by StrataQuest (scale bar = 50 µm; magnification = 20x).

Slides were labeled with the primary antibody against neurofilament, followed by the secondary, Alexa Fluor 568-conjugated antibody. After that, slides were scanned with a slide scanner at 20-fold magnification (Virtual slide microscope VS120; Olympus Europa SE & Co. KG, Hamburg, Germany). Axon quantification was performed using the imaging software, StrataQuest (Tissuegnostic, Vienna, Austria). The first step in detecting axons was to eliminate the background from images, by performing a subtraction between the original Alexa Fluor 568 marker and the gray image obtained after applying a median filter. The second step was to use the pre-processed Alexa Fluor 568 image as the input for the Dot Detection Engine. This engine detects specific, dot-like features present in a sample using a gray image as its input. Two detections were performed using different parameters, one set for the nerve fiber candidates with a high-intensity value and the other for nerve fibers with low-intensity values. These two separate detections were then combined in a single detection and used for a quantitative analysis. Figure 2A shows axons immunolabeled with anti-neurofilament and Figure 2A′ shows the same axons following automatic detection by StrataQuest.

The quantitative assessment was performed according to the two regions of interest (ROIs), i.e., the anterosuperior labrum (anterior to the LHBT origin; 1 o’clock) and the posterosuperior labrum (posterior to the LHBT origin; 11 o’clock).

### 2.3. Statistical Methods

All data obtained from the automated scanning process were analyzed by IBM SPSS® Statistics (Version 23.0, IBM Corp., Armonk, NY, USA). The Kolmogorov–Smirnov test was applied to evaluate Gaussian data distribution. If data were distributed normally, a paired *t*-test was used to test for statistical significance between anterosuperior and posterosuperior axon distribution (i.e., absolute number, area, and density). If the normality test failed, the Wilcoxon signed-rank test was performed. To determine the correlation of variables, a linear regression analysis was applied in variables with an interval measurement level (axon distribution and age). The phi coefficient was used to figure out the association of variables with a nominal level (i.e., osteoarthrosis or fracture).

A *p*-value ≤ 0.05 was regarded as statistically significant. Furthermore, descriptive histological as well as immunohistochemical statistics were performed for all study groups.

### 2.4. Ethics and Informed Consent

Prior to commencing this study, it was approved by the corresponding local ethics review board (AUVA Ethikkommission) with the approval number 06/2017. All patients signed an informed consent that they donate their intraoperatively dissected tendon tissues for scientific purposes in research. The entire study was conducted according to the guidelines for good scientific practice. The level of evidence was not applicable as this was a basic science research.

## 3. Results

Eleven LHBTs, including the superior labrum, were harvested intraoperatively. There were five right and six left shoulders from three male and eight female patients with a mean age of 73 years (range: 66–87 years). Six LHBTs were harvested from osteoarthritic shoulders dedicated to RTSA and five LHBTs were harvested from Neer grade III or IV fractures. All specimens were macroscopically healthy without signs of tendinitis, longitudinal ruptures, or degeneration like an hour-glass shape.

**Figure 3:**
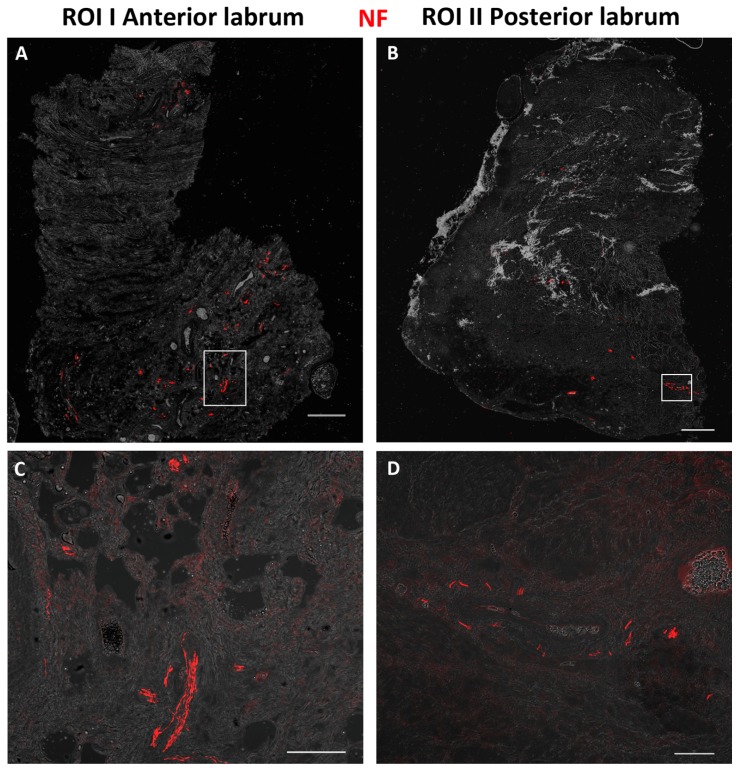
Neurofilament staining of region of interest (ROI) I and II (scale bars: (**A**,**B**) = 500 µm, (**C**) = 100 µm, (**D**) = 50 µm; magnification = 20x).

We found in all specimens, irrespective of age, gender, and baseline situation (OA vs. fracture), an inhomogeneous distribution of axons in the anterosuperior and posterosuperior parts of the labrum with high inter-individual variations (Figure 3).

**Figure 4:**
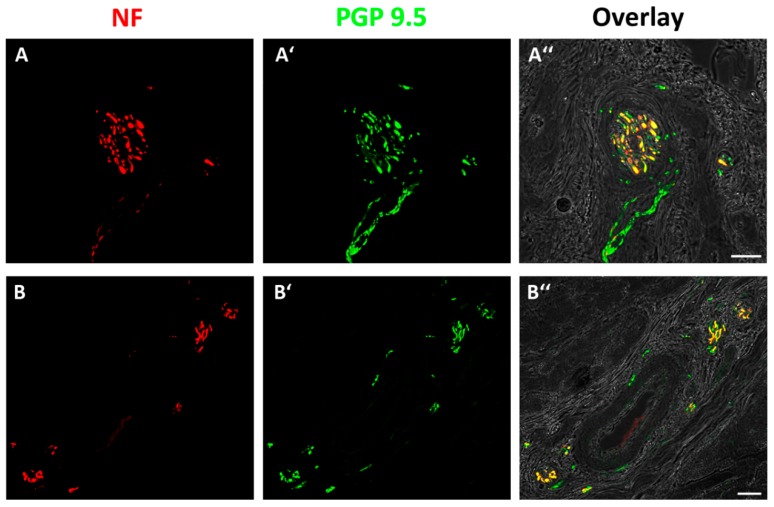
Double-labeling of NF and protein gene product (PGP) 9.5 as general markers for axons. Fluorescence images and bright field images are merged to match immuno-labeling with the anatomical structure of the tendon. (**A**,**B**) Showing nerve fibers in NF staining and (**A′**,**B′**) in PGP 9.5 staining. (**A″**,**B″**) Overlay of immuno-labeling and bright field images showing complete overlap of NF and PGP 9.5 resulting in yellow-mixed color. The bright field view shows a nerve fascicle (**A″**) and a blood vessel surrounded by axons (**B″**) (scale bars = 20 µm; magnification = 40x).

**Figure 5:**
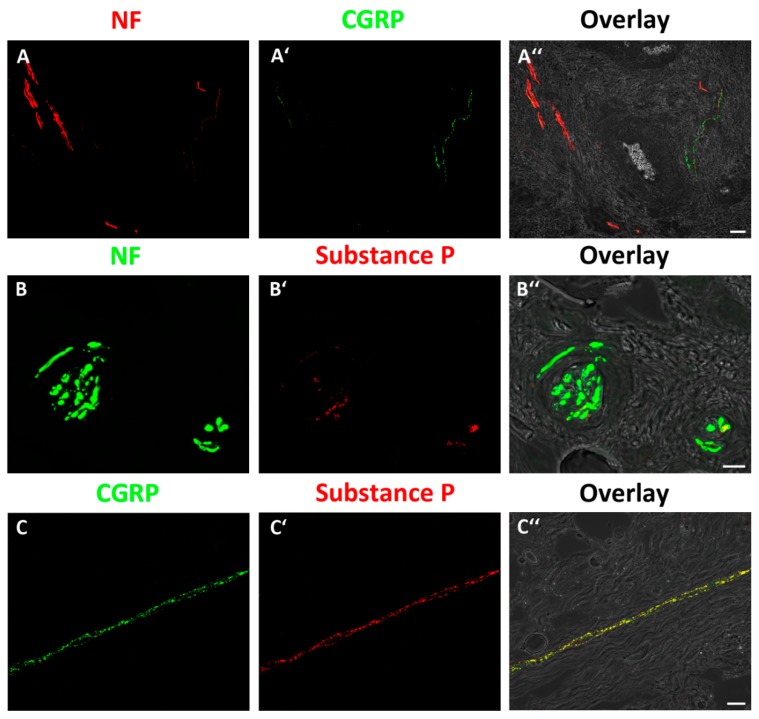
Double-labeling with NF in combination with calcitonin gene-related peptide (CGRP) (**A**–**A″**) and substance P (**B**–**B″**) as nociceptive markers as well as CGRP and substance P (**C**–**C″**). (**A″**,**B″**,**C″**) Fluorescence images and bright field images are merged to match immuno-labeling with the anatomical structure of the tendon. (**A″**,**B″**) Showing partial overlap of NF and CGRP immuno-labeling (**A″**) and NF and substance P immuno-labeling (**B″**). (**C″**) Showing co-localization of CGRP/substance P (scale bars: 5A″ = 20 µm, 5B″ = 10 µm, and 5C″ = 20 µm; magnification = 40x).

There was high expression and overlap of all used markers. The double-labeling of NF and PGP 9.5 proved the presence of axons (Figure 4A,B) and double-labeling with NF/CGRP and NF/substance P proved the presence of pain fibers (Figure 5A,B). Additional double-labeling with CGRP/substance P exhibited a co-localization of both neuropeptides (Figure 5C).

**Figure 6:**
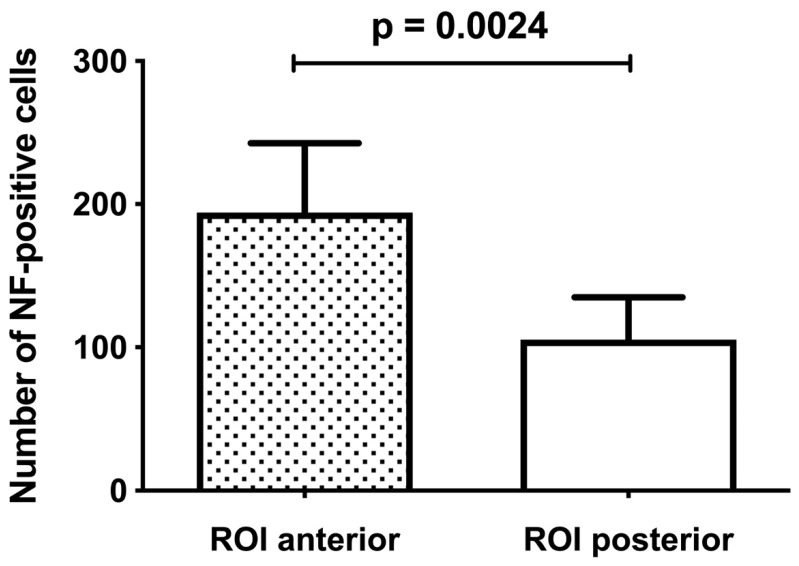
Quantitative measurement of NF-positive cells at ROI I and II. Distribution of NF-positive cells in absolute numbers revealed a significantly higher number in favor of ROI I (*p* = 0.0024).

In absolute numbers, ROI I representing the anterosuperior labrum showed a statistically significant higher number of NF-positive cells (194.2 ± 48.5) compared to ROI II (105.4 ± 29.5), representing the posterosuperior labrum (*p* < 0.01; Figure 6).

**Figure 7:**
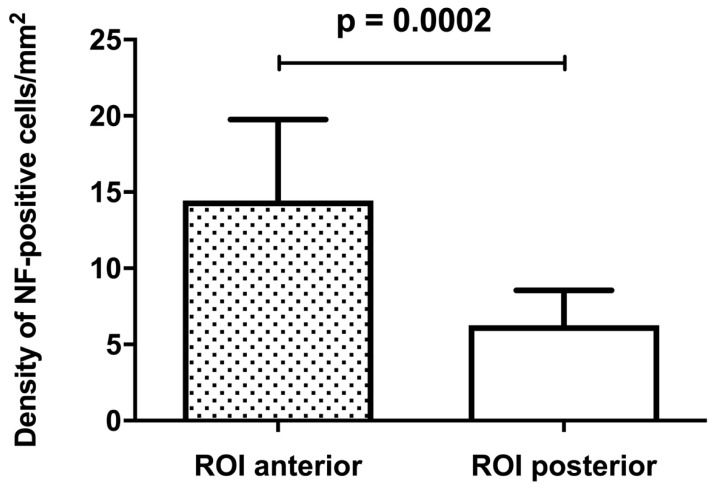
Quantitative measurement of NF-positive cells at ROI I and II. The density of NF-positive cells revealed a significantly higher density in favor of ROI I (*p* = 0.0002).

The area of NF-positive cells per square millimeters showed no significant difference (*p* = 0.85) between ROI I and ROI II (20.75 ± 2.92 vs. 20.28 ± 2.84). The density of NF-positive cells in ROI I (14.45 ± 5.32) was significantly higher compared to ROI II (6.26 ± 2.3) (*p* < 0.001; Figure 7).

The mean density of NF-positive cells was 1.5-fold higher in the anterosuperior labrum compared with the posterosuperior parts of the labrum. It was not possible to quantify the nociceptive fibers due to their small diameter (in the micrometer range). They were always found along the NF-positive axons. There was no significant difference in the absolute numbers of NF-positive cells and the density of axons when the baseline situation (OA vs. fracture) as well as gender (male vs. female) or age groups (<70 vs. >70 years) were compared.

The qualitative assessment of the specimens showed only mild signs of acute or chronic inflammation usually represented by higher vascularity or cellularity. The collagen fibers were well organized in parallel without separation showing only mild signs of degeneration.

## 4. Discussion

Concerning the variability of pain after SLAP repair, we presented data that the biceps tendon anchor is a highly innervated region with an enormous inter-individual variation independent of age, gender, or baseline situation. We found an inhomogeneous distribution of axons in the superior labrum with a significantly higher number and density in the anterior parts compared with posterior parts. Nociceptive markers were shown to overlap with the general markers for axons.

Only a few authors have analyzed neural structures in the LHBT [29,30] as it is known to act as a pain generator in various shoulder pathologies [30,31,32]. Most studies focused on the LHBT itself, but did not extend into the superior labrum [23,24,29], and analyses were only performed qualitatively [23]. So far, only one study has quantitatively assessed the distribution and density of NF in the superior labrum [25]. This study firstly investigated tissues derived from fresh specimens harvested post-mortem; secondly, the specimens were embedded in paraffin; and thirdly, only neurofilament staining worked, while the detection of CGRP and neuropeptide Y failed.

In the present study, we used intraoperatively harvested tissues and applied the techniques of cryoprotection as well as double-labeling with NF and PGP 9.5 antibodies to detect axons. Alpantaki et al. [23] described a net-like pattern of NF-positive fibers in the most proximal part of the LHBT, but we could not find this network in our specimens. In contrast, we found a cord-like fiber arrangement throughout the superior labrum. Furthermore, our results showed an association of axons with blood vessels, which is well in line with the results by Curtis et al. [33], but in contrast to the study by Alpantaki et al. [23]. Despite the specimens’ mean age of 73 years, we did not see any increased cell count or number of vessels, but some flattened and spindle-shaped nuclei, thus indicating mild degeneration.

The presence of sympathetic and sensory fibers in tendons has already been investigated [23,24,29,34,35] using different antibodies for protein S-100, neuropeptide Y, substance P, or CGRP. The last two are known to co-exist in tendons and ligaments and are involved in the process of neurogenic inflammation and vasoregulation [35]. In order to confirm the presence as well as the distribution pattern of the nociceptive markers, we performed several double-labeling experiments. This included incubation with anti-NF/anti-CGRP, anti-NF/anti-substance P, and anti-CGRP/anti-substance P. In the merged picture, the overlap of the markers was finally visualized, thus indicating the uneven distribution of nociceptors with a considerably higher amount in the anterosuperior parts of the labrum. A quantitative assessment of the nociceptive fibers, however, was not possible due to their small diameter.

Our findings of a higher and denser innervation of the anterosuperior labrum compared with the posterosuperior labrum are also supported by the anatomy of the labrum itself. Tuoheti et al. [36] described three patterns of LHBT attachment to the labrum. In all of them, the major tendinous portion of the LHBT composed of collagen fibers extended into the posterosuperior labrum, whereas the anterosuperior labrum mainly consisted of pure labral tissue. Understandably, the labral tissue contains more nerve fibers than the tendon itself.

The clinical background for this basic research study was the problem of persisting pain after SLAP repair, which is described in several studies [6,9,18,20,21]. The recommended surgical technique is anchor placement posterior to the biceps tendon anchor via the Neviaser portal [37,38] with an additional anterior anchor if the SLAP lesion extends anteriorly. The labral tissue is thereby looped by the suture, causing a strangling effect, potentially leading to nerve irritation and pain. In contrast, other techniques releasing the biceps tendon from its origin, such as tenotomy or tenodesis, are known to lead to rapid pain relief [9,39]. A very recent review article has shown that biceps tenotomy and tenodesis provide a better clinical outcome and satisfaction rate compared with repair [40]. Looking only at tenotomy versus tenodesis, tenotomy leads to better clinical results in terms of faster recovery compared with tenodesis [41,42].

Burkhart et al. [37,43] pointed out that the right position of suture anchors in SLAP repair is essential to restore stable conditions and allow proper healing. One anchor in good position has been proved to be biomechanically sufficient [44,45]. Morgan et al. [46] showed that anterior placement of the anchor has no biomechanical advantage compared to posterior placement. In a biomechanical cadaveric throwing model, McCulloch et al. [47] found that standard biceps attachment and excursion are compromised by anterior, 12 o’clock, and either side placement of the anchors leading to restricted range of motion (ROM) and even damage to the tendon.

Interestingly, Kibler and Sciascia [2] lamented in their recent systematic review that in most clinical studies, there is a lack of baseline information about the technique used, making it nearly impossible to compare clinical outcomes; 35% of the included papers did not report the number of anchors at all, and some studies used 1 or 2 anchors and others 2 to 4 anchors; and 31% did not refer to where the anchors were placed and 35% placed the anchor in a variation of 12 o’clock with sutures on either side of the biceps tendon anchor.

So far, there has been only one clinical study that investigated the clinical outcome by comparing anterior and posterior anchor placement [26]. In this study, 46 out of 49 patients were treated with an anterior and posterior anchor, whereas three patients received a posterior anchor only. Although the authors did not see any inferiority in clinical outcomes regarding anterior anchor placement, 18% of the patients underwent medical discharge from military function due to significant persistent pain, and 22% underwent subsequent biceps tenodesis for failed SLAP repair.

Taking into consideration the most recent editorial comments [15,16,17] and studies comparing different treatment options for SLAP lesions [14] as well as the results of the present study showing an inhomogeneous distribution and density of axons with a higher density of nociceptors in the anterosuperior labrum, we recommend a more cautious approach regarding the location of anchor placement in SLAP repair. A posterosuperior anchor placement seems to be beneficial for the neural structures.

The strengths of the present study are the intraoperatively harvested material, the quantitative analysis of NF-positive axons in the superior labrum, as well as double-labeling with different antibodies to increase the quality of the neural structure analysis. The limitations include the limited number of specimens and their relatively high age, which is not comparable to patients undergoing SLAP repair.

## 5. Conclusions

In conclusion, this study shows the inhomogeneous density and distribution of axons in the superior labrum with a significantly higher number and density of axons in the anterior labrum compared to the posterior parts, as evaluated by the quantitative analysis. Furthermore, we were able to prove the presence of nociceptive fibers along with other axons in the investigated specimens.
